# Physical Unclonable Function Based Privacy-Preserving Authentication Scheme for Autonomous Vehicles Using Hardware Acceleration

**DOI:** 10.3390/s26041088

**Published:** 2026-02-07

**Authors:** Rabeea Fatima, Ujunwa Madububambachu, Ahmed Sherif, Muhammad Hataba, Nick Rahimi, Kasem Khalil

**Affiliations:** 1 School of Computing Sciences and Computer Engineering, University of Southern Mississippi, Hattiesburg, MS 39406, USA; rabeea.fatima@usm.edu (R.F.); ujunwa.madububambachu@usm.edu (U.M.); nick.rahimi@usm.edu (N.R.); 2 Department of Computers and Systems, National Telecommunication Institute (NTI), Cairo 11768, Egypt; muhammad.hataba@nti.sci.eg; 3 Electrical and Computer Engineering Department, University of Mississippi, Oxford, MS 38677, USA; kmkhalil@olemiss.edu; 4 Department of Electrical Engineering, Assiut University, Assiut 71515, Egypt

**Keywords:** physical unclonable function, authentication, autonomous vehicles (AVs), hardware acceleration

## Abstract

With the rise of smart cities, technology has enabled more efficient urban management. A key part of this is the Internet of Vehicles (IoVs), which connects vehicles to smart city systems to improve transportation safety and efficiency. This integrated system enables wireless connection between vehicles, allowing for the sharing of essential traffic information. However, with all this connectivity, there are growing concerns about IoV security and privacy. This paper presents a new privacy-preserving authentication scheme for Autonomous Vehicles (AVs) in the IoV field using physical unclonable functions (PUFs). This scheme employs a bilinear pairing-based encryption technique that supports search over encrypted data. The primary aim of this scheme is to authenticate AVs inside the IoV architecture. A novel PUF design generates random keys for our authentication technique, hence boosting security. This dual-layer security strategy safeguards against a range of cyber threats, including identity fraud, man-in-the-middle attacks, and unauthorized access to personal user data. The PUF design will guarantee the true randomness of the AVs’ users’ secret keys. To handle the large amount of data involved, we use hardware acceleration with different Field-Programmable Gate Arrays (FPGAs). Our examination of privacy and security demonstrates the achievement of the defined design goals. The proposed authentication framework was fully implemented and validated on FPGA platforms to demonstrate its hardware feasibility and efficiency. The integrated heterogeneous PUF achieves an average reliability exceeding 98.5% across a wide temperature range, while maintaining near-ideal randomness with an average Hamming weight of 49.7% over multiple challenge sets. Furthermore, the uniqueness metric approaches 49.9%, confirming strong inter-device distinguishability among different PUF instances. The complete authentication architecture was synthesized on Nexys-100T, Zynq-104, and Kintex-116 devices, where the design utilizes less than 80% of slice Look-Up Tables (LUTs), under 27% of on-chip memory resources, and below 16% of DSP blocks, demonstrating low hardware overhead.

## 1. Introduction

In recent years, smart cities have undergone a massive transformation due to advancements in information and communication technologies. The Internet of Vehicles (IoVs) facilitates the collection of real-time traffic data, the control of traffic signals, and the effective distribution of traffic information, enabling the estimation of road network capacity and travel times. By processing data from IoVs, it becomes possible to improve traffic flow and ensure safe travel [1]. The transmission and management of data in the IoVs rely heavily on wireless channels, making them susceptible to threats such as information leakage and data attacks [2,3,4]. Ensuring the privacy and security of this continuously exchanged data, especially in authentication processes, is essential for maintaining system integrity and protecting user privacy in the IoVs ecosystem [5,6,7,8,9,10,11].

The increased connectivity and data interchange among vehicles cause privacy and security difficulties, which in turn pose a challenge to authentication in the IoV. Ensuring authenticity is crucial to preserving the integrity of the IoV ecosystem and protecting individual privacy, especially when wireless connectivity between vehicles increases the risk of unauthorized access and malicious data attacks. In addition, several drawbacks exist in current privacy-centric authentication frameworks for the IoV. One of these shortcomings is their vulnerability to attacks like identity fraud and data manipulation. Unfortunately, the majority of existing solutions are inadequate; they either struggle with scalability or require excessive processing resources to be feasible. The open and ever-changing nature of IoV networks may also be too much for existing solutions to manage, leaving users vulnerable to insufficient privacy protections [12,13,14,15,16,17].

In order to address these restrictions, we have developed a hardware-accelerated privacy-preserving authentication mechanism for the IoV that uses PUFs, a search-over-encrypted-data feature, and a bilinear-pairing-based encryption method. To guarantee the safe and precise transfer of vehicle data, this framework emphasizes privacy-preserving authentication. By including PUFs into key generation, an additional security layer is created, which enhances the privacy and security of IoV networks by utilizing distinct physical characteristics. This paper introduces a searchable symmetric-key encryption scheme that is suitable for both single-traffic management (like in a transportation department) and multi-user scenarios involving autonomous vehicles (AVs) in an IoV framework. In these scenarios, data, including AV identification information, is transmitted from both the traffic management system and the AVs themselves. Our authentication protocol enables the TMC and each AV to employ a single key to simultaneously encrypt AV identity data vectors (identifying vectors for all AVs overseen by a specific TMC, along with authentication requests from the AVs), while enhancing the server’s capacity to match the encrypted vectors.

A Physical Unclonable Function (PUF) is a hardware security primitive that exploits inherent manufacturing process variations to generate unique and device-specific responses to given challenges [18,19,20,21,22]. Due to uncontrollable and random physical variations introduced during fabrication, each PUF instance exhibits distinct behavior that is extremely difficult to replicate or predict, even by the original manufacturer [23,24,25]. PUFs are commonly used for lightweight key generation, device authentication, and secure identification without storing secret keys in non-volatile memory, thereby reducing vulnerability to physical and side-channel attacks. Depending on the underlying entropy source, PUFs can be broadly categorized into delay-based, memory-based (e.g., SRAM PUFs), and oscillator-based PUFs, each offering different trade-offs among reliability, entropy, and hardware cost.

To tackle the massive amounts of data generated by IoV apps and any issues with scalability, our method also uses PUFs for key generation and hardware acceleration. PUFs are developed to improve AV security mechanisms and are very difficult to replicate. They offer unmatched physical protection, low prices, ease of use, outstanding throughput, and low energy consumption, and no one else can match them. On a number of Field-Programmable Gate Array (FPGA) boards, we apply our authentication method that makes use of hardware acceleration. The feasibility of the system is demonstrated by implementing it in VHDL on an FPGA board that has several functionalities. Based on the results of our study, our approach meets all of the necessary requirements for privacy and security in design when compared to other frameworks. Furthermore, to provide an early indication of effectiveness, we summarize key experimental results obtained from both the PUF evaluation and the hardware-based authentication implementation. The proposed heterogeneous PUF demonstrates strong reliability across a wide temperature range, maintaining an average reliability exceeding 98%, while achieving near-ideal randomness and uniqueness values of approximately 49.6% and 49.8%, respectively, which are close to the theoretical optimum of 50%. These results confirm the robustness of the PUF against environmental variations and inter-device correlation. On the hardware side, the complete authentication architecture is implemented on multiple FPGA platforms, where it exhibits low computational overhead and efficient resource utilization, consuming less than 80% of LUT resources on high-capacity devices while maintaining moderate usage of DSPs and on-chip memory. The authentication latency remains sufficiently low to support real-time IoV operations, validating the practicality and scalability of the proposed design. The main contributions of this paper are summarized as follows:

- We propose a privacy-preserving authentication scheme for IoV that enables AVs to authenticate themselves securely while preventing the disclosure of sensitive vehicle identity information to the authentication server.
- We design a bilinear pairing-based searchable encryption scheme that allows AV-generated encrypted authentication requests (trapdoors) to be matched with encrypted vehicle identifiers (indices) stored at the Authentication and Data Collection Server (ADCS), ensuring confidentiality and unlinkability under an honest-but-curious threat model.
- We introduce a heterogeneous, reconfigurable PUF-based key-generation architecture that combines delay-based, ring-oscillator, and SRAM PUF components, along with response preprocessing, routing reconfiguration, and fuzzy extraction, to generate stable, high-entropy cryptographic keys for our proposed authentication scheme.
- We implement the complete authentication scheme using FPGA-based hardware acceleration and validate its feasibility across multiple FPGA platforms, demonstrating low authentication latency and efficient hardware resource utilization.
- We experimentally evaluate the proposed PUF-based design, showing strong reliability, near-ideal randomness, and high uniqueness, confirming its suitability as a hardware root of trust for IoV systems.

The subsequent sections of this paper are structured as follows. Section 2 covers the discussion of related works. Section 3 provides a detailed explanation of our system model. The proposed scheme is described in Section 4. Section 6 presents the performance evaluation. Finally, conclusive insights are provided in Section 7.

## 2. Related Work

Several schemes have been proposed to enhance the security and privacy of IoV. In [26], the authors proposed a Certificateless Aggregate Signature (CLAS) scheme to make authentication in Vehicular Ad Hoc Networks (VANETs) more efficient. Bundling signatures reduces some of the computational load. However, as the system grows and more vehicles are added, the scheme begins to falter in scalability, making it less effective in larger IoV setups. In [27], the authors explore the idea of using blockchain for authentication in the IoV. The solution relies on a public key infrastructure (PKI) to secure communication, which is highly secure. However, blockchain brings its own set of issues, like high computational costs and complexity. This can become problematic when applying it to large-scale IoV systems, where you need to keep things efficient and avoid overburdening.

The work in [28] introduces a Certificateless Short Signature Scheme (CLSS) for anonymous authentication of vehicles. It does a good job at protecting user privacy, but the bigger the system gets, the slower and less efficient it becomes. So, while it works well on a small scale, it cannot really handle the size and demands of an extensive IoV system. In [29], the authors take a different approach by using Hardware-Accelerated Authentication (HAA), relying on GPUs to speed up cryptographic processes. This helps boost performance, but the reliance on GPUs brings a hefty price tag and adds complexity to the system, which can make it harder to implement at scale in the real world.

In [30], the authors present an innovative solution for two-factor authentication in IoV systems using PUFs. Their protocol is particularly well-suited to IoV’s limited-resource environment, as it pairs PUF responses with user passwords to enable mutual authentication and minimize the risk of offline password-guessing attacks. They also introduce a Key Generation Center (KGC) to manage pseudo-identities and session keys, adding layers of anonymity and traceability to the network. This work aligns with our focus on lightweight, hardware-supported security in IoV. Still, we take it further by integrating bilinear pairing to enable more secure and efficient authentication with hardware acceleration. In [31], the authors introduce a blockchain-based authentication approach that aims to improve security and reduce latency by using decentralized consensus. Unlike traditional IoV systems that rely on a central authority, often resulting in delays, high costs, and data vulnerabilities, this method distributes data across zones managed by local controllers, reducing the need for repeated authentication as vehicles move. However, this scheme has a major scalability limitation: as more vehicles and transactions enter the network, maintaining consensus across zones can slow processing and cause network congestion, especially in busy areas. Moreover, blockchain’s high computational demands can strain IoV devices, making it challenging to deliver fast, low-latency authentication in these environments.

The study in [32] examines problems in UAV network security and offers solutions such as intrusion detection. Improving system efficiency, decreasing battery consumption, and identifying unauthorized drones using image-processing algorithms are further areas of focus. The suggested approach is not fit for purpose for real-time authentication in ever-changing IoV settings due to the high processing and latency requirements. Moreover, in contrast to our paper, it does not investigate methods that use hardware acceleration. In [33], new methods for protecting intellectual property (IP) on FPGAs are presented by the authors. The authors explain how to build an intrinsic PUF for FPGAs, examine the statistical features of PUFs based on static random-access memories (SRAMs), discuss the benefits and drawbacks of using a fuzzy extractor, and, lastly, test the design on FPGAs with embedded block RAMs. The permissioned design of Hyperledger Fabric and the consensus overhead it entails cause scalability problems for this method. Its reliance on RSU-based leader selection could also cause delays and bottlenecks in networks that support high-mobility vehicles.

Our approach, using bilinear pairing-based encryption, addresses the shortcomings of these previous schemes. First, unlike blockchain-based methods, our scheme avoids the high computational overhead, making it more practical for large-scale IoV environments. Certificateless cryptography is a good option, but it struggles with large volumes of vehicles, whereas bilinear pairing-based encryption helps manage higher volumes of authentication requests without slowing things down. In addition, using the PUFs to generate the keys required by our authentication scheme adds another layer of security. On top of that, we use FPGAs for hardware acceleration, which strikes a good balance between cost and performance. This makes our scheme ideal for large, real-time IoV systems that require fast, secure, and scalable authentication.

## 3. System Model and Design Goals

### 3.1. Network Model

**Figure 1:**
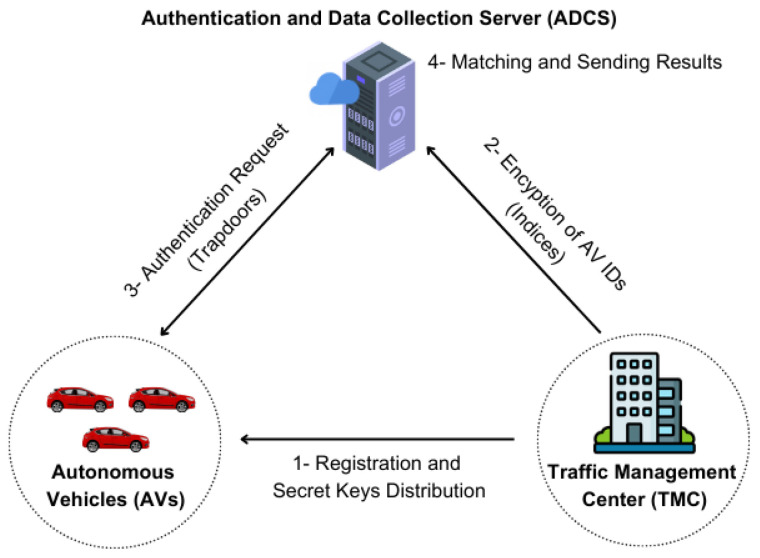
Network model.

Our proposed scheme includes three main entities: the Authentication and Data Collection Server (ADCS), Traffic Management Center (TMC), and Autonomous Vehicles (AVs), as illustrated in Figure 1. Each TMC manages a specific traffic zone and oversees AVs within it. AVs communicate with the TMC in their designated zone and transmit essential data, such as location and operational status, via the ADCS server. For secure communication, each AV must first register with the TMC before any data exchange can occur. Then, the TMC will encrypt the AVs’ IDs (indices) and send them to the ADCS server. Any AV needs first to send an authentication request (trapdoor) to the ADCS server to authenticate itself before sending its data. Finally, the ADCS server will match the trapdoor to the stored indices without knowing any sensitive AV ID information, and subsequently returns the authentication result to the requesting AV. This step completes the authentication process shown in Figure 1. The ADCS functions as a trusted third-party server known to all network participants. Its primary role is to authenticate AVs before accepting their data, without knowing any sensitive information about them, to preserve their privacy. By centralizing authentication and data handling, the ADCS ensures secure and reliable data flow across the IoV network, safeguarding real-time vehicle-to-infrastructure communication.

### 3.2. Threat Model

In our scheme, we need to account for both internal and external attackers. Our security model will be honest but curious, where some internal parties, such as the ADCS server and AVs, may follow protocols but still seek additional insight into the system, perhaps curious about details like AV IDs or encryption keys. Even though they are not malicious, their curiosity could inadvertently create security risks. On the other hand, eavesdroppers and other external attackers seeking unauthorized access to the system pose a more urgent threat. Our threat model aligns with the Dolev–Yao (DY) and Canetti–Krawczyk (CK) adversarial models [34,35], which characterize network-level and authentication-related scenarios. In order to protect user privacy within the IoV system, the proposed security goals aim to address and mitigate these risks.

### 3.3. Design Goals

Considering the aforementioned threat model, the design objectives that our proposed scheme must fulfill are as follows:

- **Scalability and Efficiency:** We need a lightweight encryption and search method that is easy to use. Data retrieval and processing incur less computational and energy expenses as a result of this. The necessary information can be safely accessed by authorized users at the same time. It also improves data encryption and retrieval across different formats and quantities, making it more flexible.
- **Authentication Query Search Over Encrypted AVs’ IDs from TMC:** For our method to work, the AV’s encrypted authentication requests must be compared with the participating TMC’s encrypted identifying data. All AVs are required to encrypt their authentication requests using the same key so that they can match all of the TMC-supplied indices for encrypted data.
- **AVs’ ID data and Authentication Query Confidentiality.** The cached AV’s encrypted identification data and authentication queries/requests do not include any sensitive or critical information that the ADCS can get.
- **Authentication Query Unlinkability.** The ADCS must be incapable of ascertaining if two authentication requests possess identical or distinct identity data.

## 4. Proposed Authentication Scheme

Key generation and distribution, AV identification data encryption, authentication query submission, and validation through encrypted data matching make up the four main components of the proposed authentication technique. Table 1 provides the main symbols of our method.

### 4.1. Key Generation

**Figure 2:**
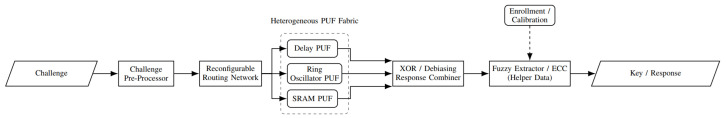
Proposed PUF architecture.

In our proposed authentication scheme, the keys are generated through our proposed PUF architecture. The proposed PUF architecture shown in Figure 2 introduces a heterogeneous, reconfigurable design that enhances unpredictability, resistance to modeling attacks, and the reliability of the generated challenge–response pairs (CRPs). The process begins with a digital challenge vector

(1) $$C=[c_{1},c_{2},\ldots ,c_{n}],$$

which passes through a challenge pre-processing unit responsible for scrambling, masking, or permuting the input bits. This preprocessing step transforms the original challenge into a new internal challenge representation $C^{\prime }=f_{\mathrm{prep}}\left(C\right)$, preventing direct correlation attacks and reducing the risk of reverse engineering. By randomizing the mapping between *C* and $C^{\prime }$, the system introduces an additional security layer before the challenge reaches the PUF fabric.

Following preprocessing, the transformed challenge $C^{\prime }$ is routed into a reconfigurable switching network that dynamically selects the path taken by the signal. This network applies a routing function

(2) $$C^{\prime \prime }=f_{\mathrm{route}}\left(C^{\prime },S\right),$$

where *S* represents the internal reconfiguration state updated periodically or during enrollment. This switching fabric enables multiple possible pathways, creating a large CRP space while mitigating overfitting in machine-learning-based attacks. The reconfigurability also ensures that identical challenges applied at different times may traverse distinct physical paths, further strengthening entropy generation.

In the proposed heterogeneous PUF fabric, each PUF primitive follows a well-defined challenge–response mechanism, tailored to its underlying physical entropy source. In the delay-based PUF, the challenge is a binary vector that configures a set of multiplexers controlling multiple signal propagation paths. Given a challenge $C_{d}\in {\left\{0,1\right\}}^{n}$, two racing signals traverse different paths determined by $C_{d}$, and the response $R_{d}\in \left\{0,1\right\}$ is generated by an arbiter that compares their arrival times. The response can be expressed as $R_{d}=\mathrm{sign}\left(\Delta t\left(C_{d}\right)\right)$, where Δt represents the delay difference between the two paths.

For the ring-oscillator (RO) PUF, the challenge $C_{r}$ selects a pair (or set) of ring oscillators from a pool of available oscillators. The response $R_{r}$ is obtained by comparing the oscillation frequencies of the selected oscillators over a fixed time window, such that $R_{r}=1$ if $f_{i}>f_{j}$, and $R_{r}=0$ otherwise. Here, the challenge effectively determines which oscillators are activated and compared, enabling a large challenge–response space while maintaining low hardware overhead.

For the SRAM PUF, the challenge $C_{s}$ corresponds to the address or block selection within the SRAM array that is read during power-up or initialization. Due to intrinsic threshold voltage variations, each SRAM cell exhibits a preferred start-up value, producing a response vector $R_{s}\in {\left\{0,1\right\}}^{m}$. In this case, the challenge does not alter circuit topology but selects which memory locations contribute to the response, enabling flexible response extraction. By combining these independent challenge–response mechanisms through a reconfigurable routing network, the proposed design exploits diverse entropy sources while maintaining a clear and well-defined challenge-response model for each PUF. This heterogeneous approach significantly increases resistance to modeling and cloning attacks compared to relying on a single PUF type.

The routed challenge signals are simultaneously fed into a heterogeneous PUF fabric consisting of three independent entropy sources: a delay-based PUF, a ring-oscillator PUF, and an SRAM-based PUF. Each component produces a raw response bit or vector, denoted by $r_{\mathrm{delay}}$, $r_{\mathrm{RO}}$, $r_{\mathrm{SRAM}}$. These responses arise from different types of manufacturing variations, path delays, oscillator frequency mismatches, and power-up memory state biases, which collectively yield complementary entropy characteristics. The three raw responses are combined through an XOR-based debiasing function,

(3) $$r_{\mathrm{comb}}=r_{\mathrm{delay}}⊕r_{\mathrm{RO}}⊕r_{\mathrm{SRAM}},$$

which suppresses structural bias, increases unpredictability, and makes modeling attacks substantially more difficult.

Finally, the combined response $r_{\mathrm{comb}}$ is processed through a fuzzy extractor that performs error correction, helper-data generation, and key reconstruction. Since environmental variations may introduce noise in the physical measurements, the fuzzy extractor applies an error-correcting code (ECC) to reconstruct a stable key. Let *w* be the noisy combined response and *h* be the stored helper data; then, the recovered secret key is obtained using

(4) K=FE(w,h),

where FE(·) denotes the fuzzy extractor reconstruction function. This produces a deterministic and reliable key despite inherent hardware noise. The enrollment block provides initial calibration, generates helper data, and stores system parameters for long-term operation. Overall, this novel PUF design combines preprocessing, reconfigurable routing, heterogeneous entropy sources, and strong post-processing to deliver a secure, robust, and machine-learning-resistant hardware root of trust.

The motivation for adopting a hybrid PUF architecture composed of three distinct PUF types, namely, delay-based, ring-oscillator, and SRAM PUFs, is to exploit complementary and independent sources of physical entropy while mitigating the inherent weaknesses of any single or dual-PUF configuration. Each PUF type is subject to different classes of attacks and reliability challenges: delay-based PUFs are susceptible to machine-learning modeling attacks, ring-oscillator PUFs may suffer from frequency aging and environmental sensitivity, and SRAM PUFs typically offer a limited challenge space. By integrating all three PUFs, the proposed design introduces orthogonal entropy sources arising from path-delay variations, oscillation-frequency mismatches, and memory-cell power-up states, respectively. From a security perspective, combining three independent PUFs significantly enlarges the effective challenge–response space and increases the complexity of constructing an accurate attack model. An adversary must simultaneously characterize multiple, statistically independent physical phenomena to predict the composite response, a task that is substantially more difficult than modeling one or two correlated PUF sources. Furthermore, even if one PUF instance becomes partially predictable or degraded over time, the remaining PUFs continue to contribute fresh entropy, preserving the overall robustness of the authentication process.

### 4.2. Key Distribution

The TMC initiates the system setup algorithm by taking the security parameter *k* as input and produces $\left\{{\mathrm{GR}}_{A},{\mathrm{GR}}_{B},{\mathrm{GR}}_{C},p,n\right\}$, where *p* is a large prime number, ${\mathrm{GR}}_{A}$, ${\mathrm{GR}}_{B}$, and ${\mathrm{GR}}_{C}$ are multiplicative groups of order *p*, and $n:{\mathrm{GR}}_{A}\times {\mathrm{GR}}_{B}\to {\mathrm{GR}}_{C}$. It also selects generators $u_{1}\in {\mathrm{GR}}_{A}$ and $u_{2}\in {\mathrm{GR}}_{B}$. Then, it uses *K*, the output of proposed PUF design. Also, it samples $R_{1}\leftarrow Z_{p}^{k\times k}$, $R_{2}\leftarrow Z_{p}^{k\times k}$ from the field $Z_{p}$. Finally, the setup algorithm outputs the public parameters $\mathrm{pub}=\left({\mathrm{GR}}_{A},{\mathrm{GR}}_{B},{\mathrm{GR}}_{C},u_{1},u_{2},p,n\right)$, and the TMC secret key $\left(TMSK\right)=\left(KR_{1},KR_{2}\right)$.

The TMC then computes the secret key of each AV (AV) in the system as follows. It samples $K^{\prime }$ and $K^{\prime \prime }$ from $Z_{p}^{k\times k}$ such that $K^{\prime }+K^{\prime \prime }=K^{-1}$ and outputs the secret key as $AVSK=\left(R_{1}^{-1}K^{\prime },R_{2}^{-1}K^{\prime \prime }\right)$. Note that for each AV to have a different secret key, $K^{\prime }$ and $K^{\prime \prime }$ are unique for each AV. Each AV then registers its ID data with the TMC.

### 4.3. Uploading Encrypted AVs’ IDs

The TMC creates a collection of encrypted indices as the encryption of AVs’ ID information using its corresponding key TMSK. For each AV (with ID ${AV}_{i}$), the TMC creates $J_{i}$ for its encrypted ID information of size *k*. The TMC samples $\gamma \leftarrow Z_{p}$ and generates the index as: $J_{i}=\left(u_{1}^{\gamma },u_{1}^{\gamma \cdot {AV}_{i}\cdot KR_{1}},u_{1}^{\gamma \cdot {AV}_{i}\cdot KR_{2}}\right)$. Then, the TMC sends $J_{i}$ to the ADCS for future use in the authentication process.

### 4.4. AV Authentication Request

For any AV that wants to send its data to the system, it first encrypts its ID ($Y_{j}$) by sampling $\delta \leftarrow Z_{p}$ and generates its trapdoor as: $U_{j}=\left(u_{2}^{\delta },u_{2}^{\delta \cdot R_{1}^{-1}K^{\prime }\cdot Y_{j}^{T}},u_{2}^{\delta \cdot R_{2}^{-1}K^{\prime \prime }\cdot Y_{j}^{T}}\right)$. Then, $U_{j}$ is sent to the ADCS for authentication and matching. When many authentication requests are sent by the same AV, the ADCS is unable to connect them. To achieve unlinkability, the sampling process (δ) must be random. It is difficult to correlate queries to the same AV because each authentication request appears different.

### 4.5. Authentication Results

Finally, the ADCS conducts a secure dot product operation between the trapdoor $U_{j}$ and each stored index $J_{i}$. The following is computed by the server:

1. $\begin{array}{l} F_{1}=n\left(u_{1}^{\gamma },u_{2}^{\delta }\right)=n{\left(u_{1},u_{2}\right)}^{\gamma \delta } \end{array}$
2. $\begin{array}{l} F_{2}=n\left(u_{1}^{\gamma \cdot {AV}_{i}\cdot KR_{1}},u_{2}^{\delta \cdot {R_{1}}^{-1}K^{\prime }\cdot Y_{j}^{T}}\right)\\ =n{\left(u_{1},u_{2}\right)}^{\gamma \delta \cdot {AV}_{i}\cdot KR_{1}{R_{1}}^{-1}K^{\prime }\cdot Y_{j}^{T}}\\ =n{\left(u_{1},u_{2}\right)}^{\gamma \delta \cdot {AV}_{i}\cdot KK^{\prime }\cdot Y_{j}^{T}} \end{array}$
3. $\begin{array}{l} F_{3}=n\left(u_{1}^{\gamma \cdot {AV}_{i}\cdot KR_{2}},u_{2}^{\delta \cdot {R_{2}}^{-1}K^{\prime \prime }\cdot Y_{j}^{T}}\right)\\ =n{\left(u_{1},u_{2}\right)}^{\gamma \delta \cdot {AV}_{i}\cdot KR_{2}{R_{2}}^{-1}K^{\prime \prime }\cdot Y_{j}^{T}}\\ =n{\left(u_{1},u_{2}\right)}^{\gamma \delta \cdot {AV}_{i}\cdot KK^{\prime \prime }\cdot Y_{j}^{T}} \end{array}$
4. $\begin{array}{l} F_{4}=F_{2}*F_{3}\\ =n{\left(u_{1},u_{2}\right)}^{\gamma \delta \cdot {AV}_{i}\cdot KK^{\prime }\cdot Y_{j}^{T}+\gamma \delta \cdot {AV}_{i}\cdot KK^{\prime \prime }\cdot Y_{j}^{T}}\\ =n{\left(u_{1},u_{2}\right)}^{\gamma \delta \cdot \langle {AV}_{i}\cdot \left(KK^{\prime }+KK^{\prime \prime }\right)\cdot Y_{j}^{T}\rangle }\\ =n{\left(u_{1},u_{2}\right)}^{\gamma \delta \cdot \langle {AV}_{i}\cdot K\left(K^{\prime }+K^{\prime \prime }\right)\cdot Y_{j}^{T}\rangle }\\ =n{\left(u_{1},u_{2}\right)}^{\gamma \delta \cdot \langle {AV}_{i}\cdot KK^{-1}\cdot Y_{j}^{T}\rangle }\\ =n{\left(u_{1},u_{2}\right)}^{\gamma \delta \cdot \langle {AV}_{i}\cdot Y_{j}^{T}\rangle }\\ =n{\left(u_{1},u_{2}\right)}^{\gamma \delta \cdot \langle {AV}_{i}\cdot Y_{j}\rangle }=F_{1}^{\langle {AV}_{i}\cdot Y_{j}\rangle }. \end{array}$
5. Knowing $F_{1}$ and $F_{4}$, we calculate $\mathrm{Discrete}\;\log(F_{4},F_{1})⟶\langle {AV}_{i}\cdot Y_{j}\rangle$ to authenticate the AV ($Y_{j}$ belonging to ${AV}_{i}$).

## 5. Security and Privacy Analysis

In accordance with the design objectives in Section 3.3, we demonstrate that our proposed scheme meets all designated capabilities and requirements by effectively addressing each design goal.

- **Scalability and Efficiency.**
  The suggested system efficiently searches encrypted IDs from the TMC and quickly addresses requests from the AVs. This is achieved by performing a dot product on the relevant encrypted IDs, using hardware acceleration. Furthermore, the computational requirements of our approach are relatively moderate. This efficiency follows directly from the construction of encrypted indices $J_{i}$ and authentication trapdoors $U_{j}$, which enable the ADCS to verify requests through secure dot product operations without decryption, as shown in Section 4.5.
- **Authentication Query Search Over Encrypted AVs’ IDs from the TMC.**
  Our proposed authentication scheme is based on searching over encrypted AV IDs, rather than traditional key-based authentication schemes, which limit exposure of sensitive identity information even to internal entities. The proposed approach employs encrypted authentication queries transmitted from an AV to the ADCS to search encrypted ID data from a participating TMC entity. Each AV utilizes a unique key to encrypt its authentication request, hence facilitating the matching of all indices supplied by the TMC for the extraction of encrypted data. Specifically, the TMC uploads encrypted indices $J_{i}$ generated using its secret key TMSK, while each AV submits a trapdoor $U_{j}$ constructed by using its secret key AVSK, enabling authentication through encrypted-domain matching rather than key-based identity verification. In addition, all sensitive communications are protected using encryption, thereby defending against eavesdropping attacks by external adversaries. So, as long as the TMC secret key (TMSK) and AV secret key (AVSK), kept secret (based on our proposed PUF design), no attackers can see or change any AV’s ID information.
- **AVs’ID data and Authentication Query Confidentiality.**
  When it comes to stored ID data or issued authentication requests, the ADCS cannot access any potentially sensitive or critical information. The AV and TMC’s encryption makes this possible. By encrypting them, IDs and authentication requests are protected from being sent to the ADCS in plaintext. By performing searches solely within the encrypted data, our scheme increases data privacy while the server refrains from decrypting it. Besides the security of the keys (generated by our proposed PUF design) used in the encryption process for the AVs’ IDs submitted by the TMC and the authentication request submitted by an AV, confidentiality is ensured also by the use of multiplicative group operations and randomization parameters γ and δ, which prevent the ADCS from recovering AVs’ identities or authentication request contents.
- **Authentication Query Unlinkability.**
  It is not possible for the ADCS to determine whether two authentication requests contain the same identification data. By leveraging the randomness of the sampling (δ), our approach ensures that authentication queries cannot be linked by generating distinct ciphertexts for the same AV ID. As each trapdoor $U_{j}$ is generated using fresh randomness, repeated authentication attempts by the same AV produce unlinkable ciphertexts.

It is important to note that the security of the proposed authentication scheme does not rely on the secrecy of the protocol itself, but rather on the secrecy of the cryptographic keys. In accordance with standard cryptographic principles, the details of the authentication scheme and its underlying algorithms may be fully known to internal or external attackers without compromising security. In the proposed framework, even if an attacker is aware of the authentication process, bilinear pairing operations, and PUF-based key generation mechanism, successful attacks remain infeasible without access to the correct secret keys. These keys are generated using the proposed PUF architecture and are never exposed in plaintext to the authentication server or to other network entities. As a result, attackers cannot encrypt valid authentication requests, decrypt stored identifiers, or link authentication queries to specific vehicles. Therefore, the overall security of the scheme fundamentally depends on the secret keys rather than on the protocol’s obscurity.

## 6. Implementation and Experimental Results

### 6.1. Hardware Architecture

**Figure 3:**
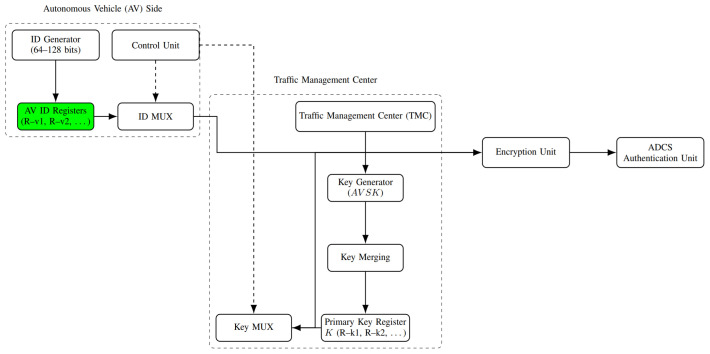
Block diagram of the proposed method.

When an AV and an ADCS exchange data, the proposed method establishes authentication. Figure 3 shows the block diagram of the suggested methodology. Every AV has its own unique identifier, which is generated internally by an ID generator. For *m* vehicles, this identifier might range from 64 bits to 128 bits. The ID generator’s output is saved in the corresponding AV register; for instance, R-v1 is associated with AV1 and R-v2 is designated with AV2. Each AV is assigned a unique key by the TMC, which is then stored in a register with *m* locations that correspond to the *m* vehicle numbers; for instance, the keys for AV1 and AV2 are kept in R-k1 and R-k2, respectively. Separate multiplexers are used to link the ID register and the key register. By utilizing the identical selection signal produced by a control unit, multiplexers transmit the required ID together with its matching key.

The registration and encryption processes of each AV are handled by the control unit. A unique identifier is created for each AV by the ID generator whenever a new AV is added to the network. The new vehicle ID is transmitted to the TMC for registration by means of a multiplexer selection signal generated by the control unit. Assume the vehicles are unchanged and that an AV must encrypt its identification data. In order to communicate with the encryption unit, the control unit generates selection signals for the ID and key multiplexers. These signals specify which AV’s ID and key are necessary.

As shown in Figure 3, the TMC is formed of many blocks that can generate new keys and encrypt an AV’s ID with a key (AVSK). The primary key *K* register stores all keys and is utilized in the hardware implementation to access the key needed for the following operations. The TMC receives an identifier (ID) from the AV during registration and stores it in a buffer until it can be sent to an ID registration unit controlled by the control unit. The control unit specifies where the new ID must be registered. The key-merging block takes all keys and merges them into a single primary key *K*. After receiving this ID, a key generator generates a new key AVSK. All of these processes are managed and coordinated by the control unit. In order to transmit the ID and key *K* to the encryption unit, the control unit uses the ID register and the key register during the encryption phase. The ADCS receives the encrypted data. Encrypted identification data received from an AV must be matched with encrypted information from the TMC as part of the ADCS task. The authentication outcome permits data acceptance if the encrypted vehicle ID corresponds to entries in the ADCS.

### 6.2. Experimental Results

#### 6.2.1. Communication Overhead

The size of the key and each index or trapdoor contribute to the communication overhead. This process makes use of a 265-bit asymmetric pairing curve (BN256) with a 32-byte group element size. An encrypted vector, that can act as either an index or a trapdoor, has a length of (2n + 1) group items, with n being the size of the ID. If the key size is 64 bits, the index or trapdoor will be 129 × 32 bytes (4128), and if it is 128 bits, it will be 257 × 32 bytes (8224). Two matrices, each holding n × n items, make up the key size for an ID size of n bits. The total key size for a 64 ID is 131,072 bytes, with 16 bytes allotted to each data item, determined by multiplying 2 × 64 × 64 × 16 bytes. The overall key size, measured in bytes, is 524,288 for a key size of 128 bits, determined as 2 × 128 × 128 × 16 bytes. Given the existing methods, these outcomes are acceptable.

#### 6.2.2. Computation Overhead

**Figure 4:**
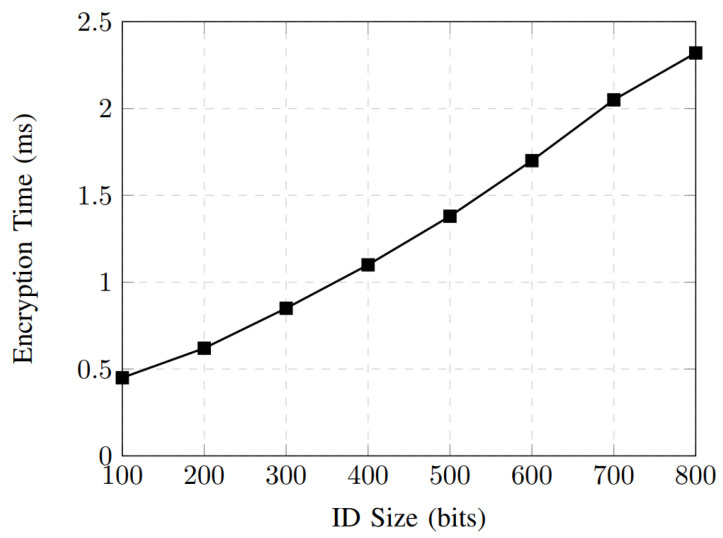
Encryption time performance.

**Figure 5:**
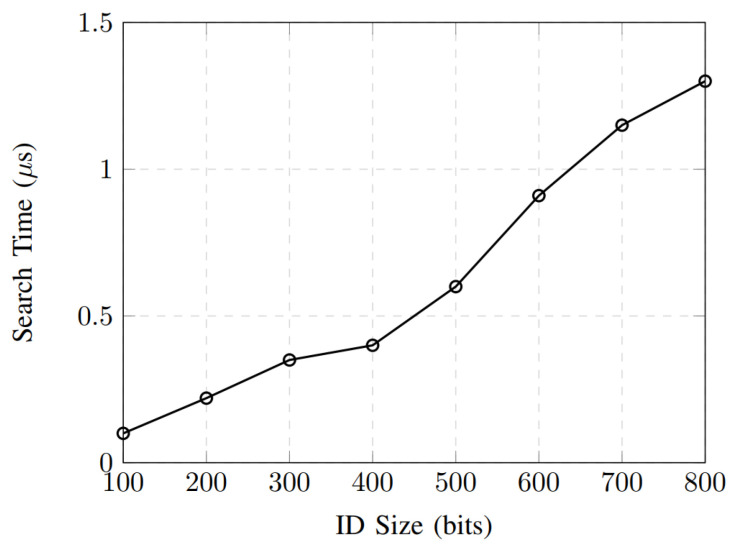
Searching time performance.

The encryption-time performance of the proposed authentication scheme is shown in Figure 4. As the AV ID size (key size as the output of the PUF) increases from 100 to 800 bits, the encryption time grows gradually and nearly linearly, rising from 0.45 ms to 2.32 ms. This behavior indicates that the encryption module’s computational complexity scales efficiently with the input size. The moderate slope of the curve demonstrates that the proposed design can accommodate larger identifier sizes without incurring a significant performance penalty, which is essential for supporting diverse security requirements in IoV environments. Moreover, the sub-millisecond encryption time for IDs up to 300 bits highlights the scheme’s suitability for real-time authentication scenarios, where low latency is a critical requirement for autonomous vehicle communications. This behavior confirms that the encryption module operates with linear time complexity, i.e., O(n), where *n* denotes the ID length. Figure 5 shows the search-time performance versus ID size for the proposed authentication scheme. The results show that the search time remains in the microsecond range even as the ID size increases, ranging from 0.10 μs for 100 bits to 1.30 μs for 800 bits. This low-latency behavior confirms the efficiency of the ID matching and lookup mechanism implemented within the authentication framework. The modest increase in search time suggests that the proposed design can support large-scale AV deployments with minimal delay, ensuring fast authentication decisions and maintaining system scalability under dense vehicular network conditions.

#### 6.2.3. Hardware Resource Utilization

**Figure 6:**
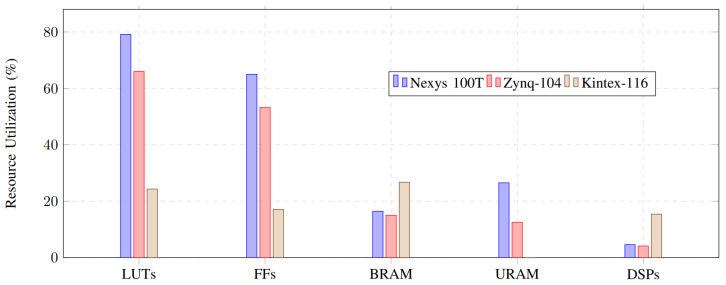
Overall hardware resource utilization of the proposed authentication scheme across different FPGA platforms.

The hardware-accelerated authentication technique is executed in VHDL across three distinct FPGAs. The hardware’s cost and performance are analyzed. The initial implementation is executed on the Nexys A7-100T FPGA, priced at $349. The second implementation is executed on the Zynq Ultrascale+ ZCU104 FPGA, priced at $1678. The Kintex Ultrascale+ 116 is the third FPGA, with a valuation of $3882. We used the same methodology across varying FPGA prices and resources and conducted a performance and cost comparison. Resource usage is assessed for each FPGA implementation. We quantified the resources utilized by the authentication method, and the ratio of utilized resources to available resources is shown in Figure 6. The Kintex 116 FPGA accommodates substantial ID sizes (an elevated security level) and offers greater resources than alternative FPGAs for the authentication method; nonetheless, it incurs a higher cost. The Nexys 100T FPGA is utilized for modest ID sizes (low-security level) due to its limited resources and low cost. The mid-range is implemented on the Zynq104 FPGA due to its moderate performance and affordability. This study showcases many uses of the hardware-accelerated authentication mechanism to facilitate diverse AV resources in the IoV era.

Although the proposed heterogeneous PUF fabric integrates three independent entropy sources, namely a delay-based PUF, a ring-oscillator PUF, and an SRAM-based PUF, the overall hardware cost remains practical and well within the capabilities of modern FPGA platforms. Each PUF primitive is lightweight by design: the delay-based PUF relies primarily on configurable routing and simple arbiters; the ring-oscillator PUF uses a limited number of inverters and counters; and the SRAM PUF leverages existing on-chip memory blocks without requiring additional custom circuitry. As demonstrated by the FPGA synthesis results, the complete authentication architecture, including the heterogeneous PUF fabric, consumes a modest portion of logic and memory resources, with DSP utilization remaining below 16% and memory usage below 27% across evaluated platforms. Furthermore, the PUF components are not operated concurrently; instead, the reconfigurable routing network selectively activates individual PUF instances, which further reduces dynamic power consumption and hardware overhead. These characteristics confirm that the proposed heterogeneous PUF is feasible for practical deployment and offers a favorable trade-off between hardware cost and security enhancement in real-world autonomous vehicular systems.

#### 6.2.4. PUF Performance Analysis

The results summarized in Table 2 demonstrate that the proposed heterogeneous PUF design achieves strong statistical performance across the most critical security metrics. The uniqueness metric, measured at 49.2%, is very close to the theoretical ideal of 50%, indicating that PUF instances from different chips produce highly distinguishable responses. Likewise, the reliability measures show that the design delivers stable response bits across environmental variations, including temperature, voltage, and aging, achieving 97.8% stability. These metrics confirm that the joint operation of the reconfigurable routing network and the mixed PUF fabric effectively reduces noise and path imbalance.

The remaining statistical indicators, bit-aliasing, uniformity, entropy, and steadiness, also reinforce the robustness of the design. The uniformity and bit-aliasing results, at 49.8% and 50.6%, respectively, suggest that the PUF output is unbiased and does not favor specific bit positions across chips. The entropy level of 0.994n shows that the combined PUF elements, when processed through the XOR/combiner and fuzzy extractor, generate nearly ideal randomness. Furthermore, the steadiness score of 98.5% after fuzzy extraction indicates that the error-correction subsystem successfully mitigates instability arising from individual PUF components. Together, these results validate the strength of the proposed architecture in providing secure, stable, and high-quality challenge–response behavior.

**Figure 7:**
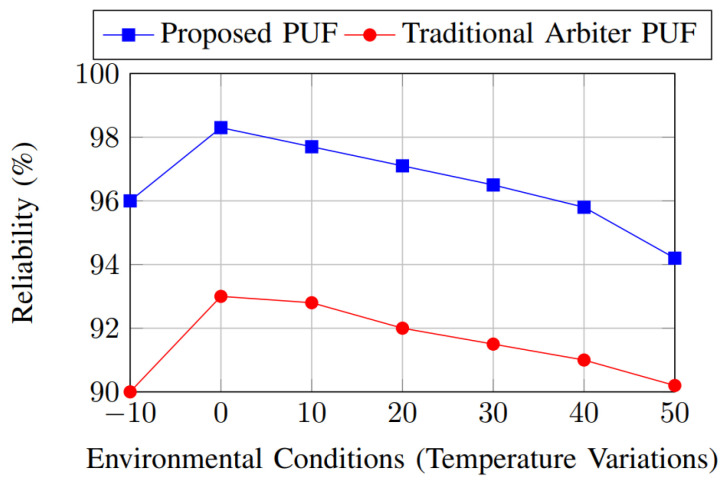
Reliability comparison.

Figure 7 illustrates the reliability comparison between the proposed PUF and a traditional PUF across a wide temperature range. Reliability is defined as the probability that the same response is reproduced under varying environmental conditions for a fixed challenge. As observed, the proposed PUF maintains consistently higher reliability as temperature deviates from the nominal operating point. This improvement is mainly attributed to the heterogeneous PUF fabric and the response stabilization stage, which mitigates temperature-induced delay variations and metastability effects. Furthermore, incorporating a fuzzy extractor with error correction ensures that minor bit flips caused by thermal noise are corrected, leading to near-ideal reliability even at elevated temperatures. In contrast, the traditional PUF exhibits a noticeable degradation in reliability as temperature increases, highlighting its sensitivity to environmental variations.

**Figure 8:**
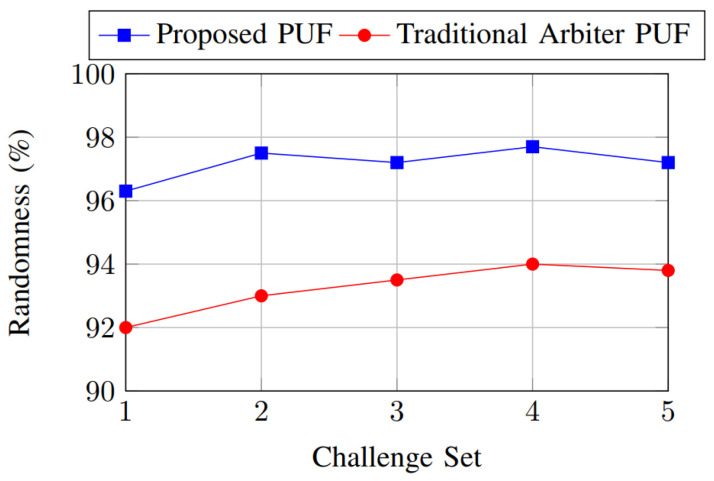
Randomness comparison.

The randomness comparison shown in Figure 8 evaluates the statistical balance of the PUF responses across different challenge sets. Randomness is measured as the ratio of logic ‘1’s to the total response length, with an ideal value close to 50%. The proposed PUF demonstrates randomness that remains tightly centered around the ideal value for all evaluated challenge sets, indicating strong resistance to bias. This behavior is achieved through the combined effects of reconfigurable routing, heterogeneous PUF cores, and XOR-based response combining, which collectively decorrelate structural dependencies within individual PUF primitives. In comparison, the traditional PUF exhibits larger fluctuations in randomness across challenge sets, suggesting greater susceptibility to systematic bias and correlated responses.

**Figure 9:**
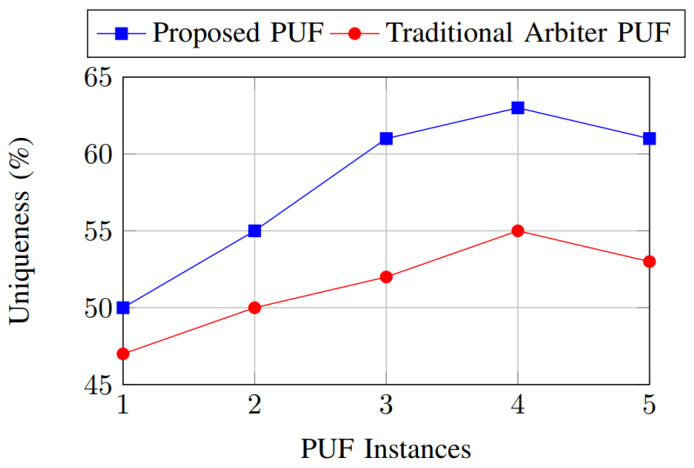
Uniqueness comparison.

Figure 9 presents the uniqueness results for the proposed and traditional PUFs as the number of evaluated PUF instances increases. Uniqueness is quantified as the average inter-device Hamming distance between responses to identical challenges, with an ideal value of 50%. The proposed PUF achieves unique values that closely approach the ideal target across the entire PUF set, demonstrating substantial inter-chip variability. This improvement is primarily due to the heterogeneous nature of the PUF fabric, which amplifies manufacturing variations across multiple physical entropy sources. Additionally, the reconfigurable routing network further enhances diversity by altering signal paths on a per-instance basis. In contrast, the traditional PUF exhibits lower and less stable uniqueness values, indicating reduced resistance to device cloning and modeling attacks.

The randomness of the proposed PUF is evaluated using the average Hamming weight, which measures the balance between logic ‘0’ and logic ‘1’ in the generated responses. For an *n*-bit PUF response $R=\{r_{1},r_{2},\ldots ,r_{n}\}$, the Hamming weight is defined as $\mathrm{HW}\left(R\right)=\sum_{i=1}^{n}r_{i}$, and the normalized average Hamming weight [36] is expressed as

(5) $${\mathrm{HW}}_{\mathrm{avg}}=\frac{1}{n}\sum_{i=1}^{n}r_{i}\times 100\%.$$

Experimental evaluation of the proposed design over multiple challenge sets yields an average Hamming weight of 49.7%, which is close to the ideal value.

Table 3 presents a quantitative comparison between the proposed PUF-based authentication method and several recent state-of-the-art designs in terms of uniqueness, reliability, and uniformity. The proposed method achieves a uniqueness of 49.2%, which is very close to the ideal value of 50% and comparable to the best-performing prior work, indicating strong inter-device distinguishability. In terms of reliability, the proposed design outperforms existing schemes, achieving 97.8% and demonstrating robust, stable response generation under environmental and operating variations. Furthermore, the uniformity of the proposed method reaches 49.8%, closely approximating the ideal distribution and improving upon [37], while maintaining comparable performance to [38]. Overall, the results confirm that the proposed approach provides a well-balanced improvement across all key PUF metrics, making it more suitable for secure, reliable authentication in resource-constrained, safety-critical IoV environments.

## 7. Conclusions

With the rise of IoV services, ensuring security and privacy has become essential. This paper introduces a novel privacy-preserving authentication mechanism for AVs within the IoV domain, utilizing physical unclonable functions (PUFs). This strategy uses a bilinear-pairing-based encryption method to facilitate search within encrypted data. The principal objective of this scheme is to verify the authenticity of AVs inside the IoV framework. A revolutionary PUF architecture produces random keys for our authentication method, hence enhancing security. This dual-layer security approach protects against many cyber risks, such as identity theft, man-in-the-middle attacks, and unauthorized access to confidential user information. The PUF design will ensure the genuine randomization of the secret keys for AV users. To manage the substantial volume of data, we employ hardware acceleration via FPGAs. Our security and privacy evaluation demonstrates that the scheme effectively authenticates data sources without revealing any sensitive information. Moreover, by using hardware acceleration, we achieved substantial reductions in computational and communication overhead, making this solution both efficient and practical for real-time IoV needs.

## Figures and Tables

**Table 1:** Main Notations.

Symbol	Description
PUF	Physical Unclonable Function.
AV	Autonomous Vehicle.
TMC	Transport Management center.
ADCS	Authentication and Data Collection Server.
*k*	Security parameter.
*p*	Large prime number.
*n*	Bilinear pairing function.
${\mathrm{GR}}_{A},{\mathrm{GR}}_{B},{\mathrm{GR}}_{C}$	Multiplicative groups of order *p*.
TMSK	TMC secret key.
AVSK	AV secret key.

**Table 2:** Evaluation Results of the Novel Heterogeneous PUF.

Metric	Expected Ideal	Measured Value	Interpretation
Uniqueness (%)	50	49.2	Excellent inter-chip variation
Reliability (%)	100	97.8	Stable responses under noise
Bit-Aliasing (%)	50	50.6	No structural bias across bits
Uniformity (%)	50	49.8	Balanced 0/1 distribution
Entropy (bits)	*n*	0.994*n*	Near-ideal randomness per bit
Steadiness (%)	100	98.5	Strong resilience after ECC

**Table 3:** Comparison with existing works.

Ref.	Uniqueness	Reliability	Uniformity
[37]	45.25	95.93	48.30
[38]	49	85.95	50
[39]	41.53	95.50	N/A
The proposed method	49.2	97.8	49.8

## Data Availability

The data presented in this study are available on request from the corresponding author.
